# Acetone Extract from *Rhodomyrtus tomentosa*: A Potent Natural Antioxidant

**DOI:** 10.1155/2012/535479

**Published:** 2012-10-18

**Authors:** Goodla Lavanya, Supayang Piyawan Voravuthikunchai, Nongporn Hutadilok Towatana

**Affiliations:** ^1^Natural Products Research Centre, Prince of Songkla University, Hat Yai, Songkhla 90112, Thailand; ^2^Department of Microbiology, Prince of Songkla University, Hat Yai, Songkhla 90112, Thailand; ^3^Department of Biochemistry, Prince of Songkla University, Hat Yai, Songkhla 90112, Thailand

## Abstract

*Rhodomyrtus tomentosa* (Myrtaceae) has been employed in traditional Thai medicine to treat colic diarrhoea, dysentery, abscesses, haemorrhage, and gynaecopathy. In addition, it has been used to formulate skin-whitening, anti-aging and skin beautifying agents. Ethnomedical activities of this plant may be due its antioxidant property. Hence, the aim of this study was to evaluate both *in vitro* and *in vivo* antioxidant activities of *R. tomentosa* leaf extract. *In vitro* antioxidant activity of the extract was assessed by lipid peroxidation inhibition capacity, ferric reducing antioxidant power, and metal chelating activity. *R. tomentosa* extract demonstrated its free radical scavenging effects in concentration dependent manner. *In vivo* antioxidant activity of the extract was conducted in Swiss *Albino* mice. Levels of thio-barbituric acid reactive substances (TBARS), glutathione (GSH), and the activities of antioxidant enzymes including superoxide dismutase (SOD), catalase (CAT), and glutathione peroxidase (GPx) in blood, liver, and kidney were analyzed using microtitre plate photometer. Administration of CCl_4_ caused significant increase in TBARS and decrease in GSH, SOD, CAT and GPx levels. In contrast, *R. tomentosa* extract (0.8 g/kg) effectively prevented these alterations and maintained the antioxidant status. The results suggest that *R. tomentosa* extract can serve as a potent antioxidant.

## 1. Introduction

Oxidative stress is believed to be one of the major causes of more than hundred types of human diseases as it can result in severe cellular dysfunction due to peroxidation of membrane lipids, protein modification, depletion of nicotinamide nucleotides, rises in intracellular free calcium ions (Ca^2+^), cytoskeletal disruption, and DNA damage [1]. Antioxidants are the molecules that can delay or prevent the oxidation of cellular substrates [2]. Natural antioxidants mainly derived from plants are preferred over synthetic antioxidants, as many of them including propylgallate, citric acid, butylated hydroxyanisole, and butylated hydroxytoluene are suspected to be hepatotoxic and carcinogenic [3].

*Rhodomyrtus tomentosa* (Aiton) Hassk. (Family Myrtaceae) is an ornamental, evergreen shrub grows up to four meters tall. This plant species is native to southern and southeastern Asia [4]. It has been often used in traditional Thai medicine to treat colic diarrhoea [5], dysentery, abscesses, haemorrhage, and gynecopathy [6]. In addition, it is used to formulate skin-whitening, antiaging, and skin-beautifying agents [7]. The extract from this plant possess strong inhibitory activity against gram-positive bacteria [8, 9]. A range of compounds including acylphloroglucinol, flavonoids, tannins, and triterpenes have been identified from this plant [10]. Rhodomyrtone, an acylphloroglucinol component from this plant, has been recently reported as an effective anti-infective agent against *Streptococcus pyogenes *[11], *Staphylococcus aureus,* and biofilm-forming Staphylococci [12].

 Although *Rhodomyrtus tomentosa* extract has been extensively investigated for its antimicrobial properties [13–16], less or no data regarding its antioxidant properties are available. It is believed that some of the ethnomedical and reported biological activities of this plant may be due its antioxidant property. Hence, the present study was aimed to evaluate both *in vitro* and *in vivo* antioxidant activities of the extract from *Rhodomyrtus tomentosa* leaves.

## 2. Material and Methods

### 2.1. Chemicals

Thiobarbituric acid (TBA), 2,4,6-tripyridyl-s-triazine (TPTZ), and ferrozine were purchased from Sigma-Aldrich Co., USA. Gallic acid, ellagic acid, and olive oil were obtained from Fluka Chemie GmbH CH-9471 Buchs, Spain. Reagents for measuring Thio-barbituric acid reacting substances (TBARS), total glutathione (GSH), superoxide dismutase (SOD), catalase (CAT), and glutathione peroxidase (GPx) were purchased from Cayman Chemical Company, USA. All other chemicals used were analytical grade.

### 2.2. Plant Material

*R. tomentosa* leaves were collected from the Singha Nakorn District, Songkhla Province in the southern part of Thailand during February 2010. The voucher specimen (A. Hiranrat 001) was identified by J. Wai and has been deposited in the herbarium of the Department of Biology, Faculty of Science, Prince of Songkla University, Thailand.

### 2.3. Extract Preparation

*R. tomentosa* leaves were dried in oven at 60°C for 48 h and grounded in an electric blender. The powder was extracted with 95% acetone and left at room temperature for 7 days. The extract was evaporated by using a rotary evaporator (BUCHI Rotavapor R-114, Buchi Labortechnik AG, Flawil, Switzerland) and kept at 4°C.

### 2.4. *In Vitro* Studies

#### 2.4.1. Lipid Peroxidation Inhibitory Assay

Lipid peroxidation induced by Fe^2+^ in a known concentration of liposome was estimated as TBARS according to Ohkawa's method [17]. The reaction mixture contained liposome from soyabean phosphotidylcholine 0.1 mL (0.2 g/L) in Tris-HCl buffer (20 mM, pH 7.0), KCl (30 mM), FeSO_4_(NH_4_)_2_SO_4_ (0.06 mM), and various concentrations of *R. tomentosa *extract in a final volume of 0.5 mL. After the reaction mixture was incubated at the 37°C for 1 h, 0.4 mL of the reaction mixture was treated with 0.2 mL sodium dodecyl sulfate (SDS, 8.1%), 1.5 mL TBA (0.8%), and 1.5 mL acetic acid (20%, pH 3.5). The total volume was made up to 4.0 mL with distilled water and then kept in a water bath at 95 to 100°C for 1 h. After cooling, 1.0 mL of distilled water and 5.0 mL of n-butanol and pyridine mixture (15 : 1 v/v) were added to the reaction mixture, shaken vigorously and centrifuged at 1912 *g* for 10 min (SORVALL RC5C PLUS centrifuge, USA). The butanol-pyridine layer was removed, and its absorbance at 532 nm was measured to quantify TBARS. Inhibition of lipid peroxidation was determined by comparing the optical density of samples with that of standard curve of gallic acid.

#### 2.4.2. Ferric Reducing Antioxidant Power (FRAP)

FRAP was performed according to Erel's method [18] with slight modifications. Briefly, the working FRAP reagent was prepared by mixing 300 mM acetate buffer (pH 3.6), 10 mM TPTZ in 40 mM HCl solution, and 20 mM FeCl_3_.6H_2_O in a 10 : 1 : 1 ratio, just before use and heated to 37°C. A total of 150 *μ*L working FRAP reagent was added to each well in a 96-well microtiter plate. A blank reading was taken at 593 nm using a micro titre plate reader (ELX 808, BioTek Instruments, Inc. USA). A total of 20 *μ*L of the sample (1 g/L of plant extract), and the standards (gallic and ellagic acids) were then added to wells containing FRAP reagent. The mixture was then incubated at 37°C for 10 min, and the absorbance was measured at 593 nm for second time. The change in absorbance from the initial blank reading was then compared with a standard curve. Standards were run at different concentrations (25, 50, 75, 100, 200, 400, 600, 800, and 1000 *μ*M). A standard curve was then prepared by plotting FRAP value of each standard versus its concentrations. The values were expressed as mM of gallic acid and ellagic acid equivalents per 1 mg extract.

#### 2.4.3. Chelating Activity on Ferrous Ions (Fe^2+^)

Fe^2+^ chelating activity was measured by inhibition of the formation of Fe^2+^-ferrozine complex after treatment of test material with Fe^2+^, following Dinis method [19]. Fe^2+^-chelating ability of *R. tomentosa* extract was monitored by the absorbance of the ferrous ion-ferrozine complex at 562 nm. Briefly, different concentrations of *R. tomentosa* extract (20–100 *μ*g/mL) in 1% DMSO (0.4 mL) were added to a solution of 0.6 mM FeCl_2_ (0.1 mL). The reaction was initiated by the addition of 5 mM ferrozine (0.1 mL) dissolved in methanol. Then the mixture was shaken vigorously and left at room temperature for 10 min. Absorbance of the solution was then measured spectrophotometrically (Perkin Elmer Lambda25 UV/VIS spectrometer) at 562 nm. The control contains 1% DMSO, FeCl_2_, and ferrozine. The ferrous ion-chelating effect of *R. tomentosa* extract was determined as EDTA equivalent.

### 2.5. *In Vivo* Studies

#### 2.5.1. Experimental Animals

 Forty Swiss albino mice (*Mus musculus*) weighing 28–35 g of the male sex were obtained from the Laboratory Animal Facility Unit, Faculty of Science, Prince of Songkla University, Hat Yai, Thailand. They were housed in an identical wire-mesh-bottomed stainless-steel cages containing six mice per cage and maintained in an air-conditioned room at 25 ± 2°C, 50 to 60% relative humidity and artificial illumination between 06:00 and 18:00 h. Commercial diets (C.P. Mice Feed, Charoen Phokphand Group, Bangkok, Thailand) and drinking water were provided *ad libitum*. All procedures concerning animal treatments and experimentations in this study were reviewed and approved by the Institutional Committee for Ethical Use of Experimental Animals at Prince of Songkla University.

#### 2.5.2. Experimental Design

*In vivo* antioxidant activity of *R. tomentosa* leaves extract was determined by using carbon tetrachloride (CCl_4_)-induced oxidative stress in the mouse model. The animals were divided into six groups of six animals each. Before treatment, the mice were fasted overnight, but with free access to water. Group I served as control which received 1% DMSO (10 mL/kg/day, intragastric gavage (ig)) for 14 days. Group II served as the toxin control and received CCl_4_ alone. Group III served as positive control and received *α*-tocopherol (0.1 g/kg/day, ig) for 14 days. Group IV to VI served as treated group and received 0.2, 0.4, and 0.8 g/kg body weight of *R. tomentosa* extract in 1% DMSO (10 mL/kg/day, ig), respectively, for 14 days. Except Group I, all other groups received subcutaneous injection of 30% CCl_4_ in olive oil (10 mL/kg, once in each 72 h of 14 days). All animals had free access to diet and drinking water during the study. The animals were sacrificed on day 15 by cervical decapitation. Blood samples were collected from the heart puncture into an anticoagulant-containing tube to prevent the clot. The liver and kidney were quickly harvested and weighed. Blood and tissue samples were stored at −80°C for further use.

#### 2.5.3. Assessment of the Extent of Oxidative Stress in Blood and Tissues

 The level of TBARS and GSH, the activities of total SOD, CAT, and GPx in blood and tissues were quantified using commercially available kits, purchased from Cayman Chemical Company, USA, following the manufacturer's instructions. Briefly, Liver and kidney were homogenized (10% homogenate) in different assay buffers as per requirement of the assay, and the homogenates were centrifuged at 10,000 ×g for 15 min at 4°C (Sorvall RC5C PLUS centrifuge, USA). The obtained supernatants collected to determine the levels of TBARS and antioxidants according to the kit protocol. All the assays were performed by using a micro titer plate photometer (BioTek Instruments, Inc., USA).

### 2.6. Statistical Analysis

All the values are represented as mean ± SEM. Data for *in vitro* experiments were analyzed by linear regression analysis and *t*-test. Data on biochemical investigations of *in vivo* experiments were analyzed by one-way analysis of variance (ANOVA), and the group means were compared by dunnet's multiple range Test (DMRT). A probability of *P* < 0.05 was considered as significant.

## 3. Results and Discussion

### 3.1. *In Vitro* Antioxidant Activity

#### 3.1.1. Lipid Peroxidation Inhibitory Assay

**Figure 1:**
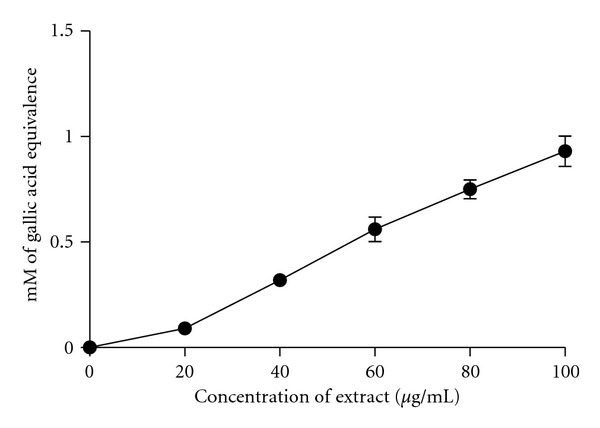
Inhibition of lipid peroxidation at different concentrations of *R. tomentosa* extract. The values are expressed as gallic acid equivalence (mean ± SEM; *r* ^2^ = 0.996, *P* < 0.0001). *n* = 6 pairs.

The inhibitory effect of acetone extract from *R. tomentosa* leaves on FeCl_2_-ascorbic acid-induced malondialdehyde (MDA) production in liposome system is represented in Figure 1. Lipid peroxidative degradation of the biomembrane is one of the principal mechanisms for the generation of free radicals [20]. The level of lipid peroxidation was determined as thiobarbituric acid reactive substances, which are mainly aldehydes and 99% TBARS is malondialdehyde [21]. The rate of inhibition of MDA formation increased as the concentration of extract increased. *R. tomentosa* extract was able to significantly inhibit the generation of lipid peroxides (*P* < 0.0001). The lipid peroxidation inhibition capacity of the extract was equal to 0.93 ± 0.07 mM gallic acid, at 100 *μ*g/mL.

#### 3.1.2. Ferric Reducing Antioxidant Power

**Figure 2:**
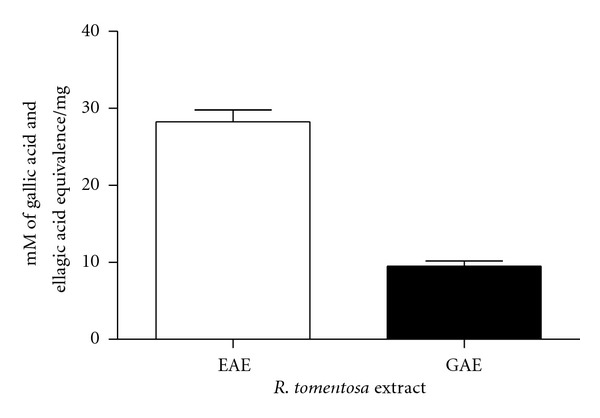
Ferric ion reducing power of *R. tomentosa* extract at 1 mg/mL concentration. Values are expressed as gallic acid (GAE), and ellagic acid (EAE) equivalence (mean ± SEM; *r* ^2^ = 0.969, *P* < 0.0004). *n* = 2 in triplicates.

 The reducing capacity of a compound may serve as a significant indicator of its potential antioxidant activity [22]. For the measurement of reductive ability of *R. tomentosa* extract, the transformation of Fe^3+^-Fe^2+^ was investigated in its presence. The extract showed a rapid and increased tendency to reduce Fe^3+^-Fe^2+^ as equal as 10.8 ± 1.12 mM gallic acid and 30.5 ± 5.22 mM ellagic acidcals, respectively, at 1 mg/mL (Figure 2). The extract exhibited strong reducing ability of 2.7-fold and 3.0-fold higher than that of gallic acid and ellagic acid, respectively.

#### 3.1.3. Chelating Activity on Ferrous Ions

**Figure 3:**
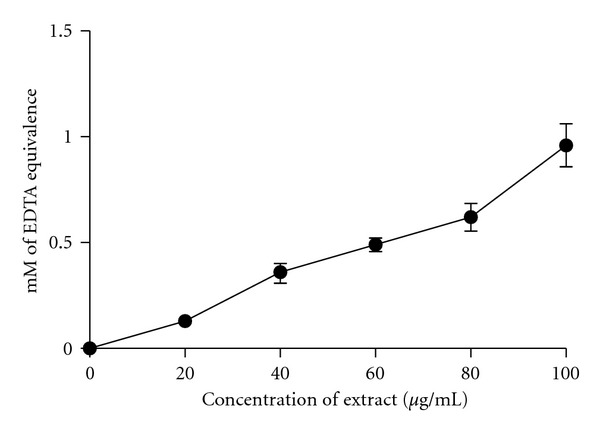
Metal ion (Fe^2+^) chelating activity at different concentrations of *R. tomentosa* extract. Values are expressed as EDTA equivalence (mean ± SEM; *r* ^2^ = 0.9714, *P* < 0.0001). *n* = 6 pairs.

Transition metal ions such as ferrous ions are known to catalyze the formation of free radicals, and a minority of metal ions could accelerate the lipid peroxidation [23] by decomposing lipid hydroperoxides into peroxyl and alkoxyl radicals that can themselves abstract hydrogen and perpetuate the chain reaction of lipid peroxidation [24]. Ferrozine, a competitive metal ion chelate, can quantitatively form complexes with Fe^2+^. In the presence of chelating agents, the complex formation is disrupted; with the result, the red colour of the complex is decreased. Measurement of colour reduction, therefore, allows estimation of the chelating activity of the coexisting chelate [25]. Acetone extract from *R. tomentosa* and EDTA (a potent metal ion-chelating agent) interfered with the formation of ferrous-ferrozine complex, suggesting that they are capable to capture ferrous ion before ferrozine. Liner decrease in the absorbance of Fe^2+^-ferrozine complex was observed, with the increase of plant extract concentration (Figure 3). The metal ion chelating activity of *R. tomentosa* extract is equal to 0.96 ± 0.10 mM EDTA, at 100 *μ*g/mL. The antioxidant activity of putative antioxidants has been attributed to various mechanisms, among which are prevention of chain initiation, binding of transition metal ion catalysts, decomposition of peroxides, prevention of continued hydrogen abstraction, reductive capacity, and radical scavenging capability [26]. The radical scavenging capability of the extract may be due to the synergistic effect of the phytochemicals present in it.

### 3.2. *In Vivo* Antioxidant Activity

Preliminary results from *in vivo* acute toxicity test elucidated that the acetone extract of *R. tomentosa *is safe when orally administered at a concentration of 2 g/kg body weight. *In vivo* antioxidant activity of *R. tomentosa* leaf extract was carried out at doses of 0.2, 0.4, and 0.8 g/kg body weight in mice induced with CCl_4_. Carbon tetrachloride is a well-established hepatotoxin and used by various researchers, for experimental induction of oxidative stress [27]. CCl_4_ toxicity depends on the reductive dehalogenation of CCl_4_ catalyzed by Cytochrome P_450_ in endoplasmic reticulum leading to the generation of an unstable complex trichloromethyl (CCl_3_^●^) radical, which initiates a cascade of free radical reactions resulting in an increase of lipid peroxidation and a reduction in some enzyme activities [28]. A number of recent reports clearly demonstrated that in addition to hepatic problems, CCl_4_ also causes disorders in kidneys, lungs, testis, and brain as well as in blood by generating free radicals [29, 30]. Lipid peroxidation and levels of endogenous antioxidants are commonly used index to assess the oxidative stress. GSH, SOD, CAT, and GPx from the primary team of defense against reactive oxygen species [31]. *In vivo* antioxidant activity of the extract was determined by analyzing the levels of TBARS and endogenous antioxidants in the blood, liver, and kidneys of experimental animals. MDA is the end product of lipid peroxidation in membrane fatty acids representing oxidative damage caused by free radicals resulting in structural modification of membrane with the release of cell and organelle contents, loss of essential fatty acids in the production of cytosolic peroxide products [32]. The tripeptide, GSH, is the most important cellular defense mechanism that exists in the cell. The ability of the cell to regenerate GSH is an important factor in the efficiency of managing oxidative insults. A depletion of intracellular GSH has been reported during oxidative stress conditions, where there is an increase in ROS [33].

**Figure 4:**
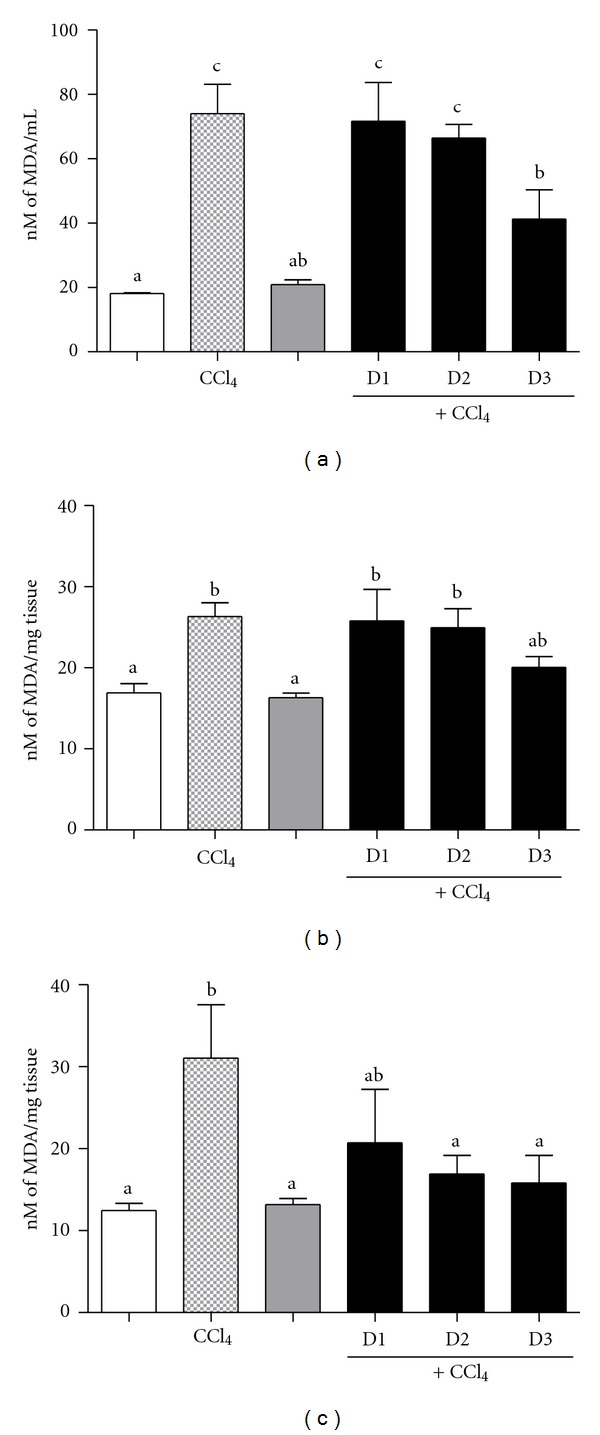
Levels of thiobarbituric acid reacting substances (TBARS) in blood (a), liver (b), and kidneys (c) of mice in each group. TBARS was measured as nM of malondialdehyde formed. Each data bar represents mean of six replicates in each group ± SEM. Means having same alphabets on each bar do not differ (*P* ≤ 0.05) from each other. Negative control group: receive 1% DMSO (White column), toxin control group: receive 30% CCl_4_ (Light gray column), positive control group: receive *α*-tocopherol + CCl_4_ (Heavy gray column), and extract treated groups: received three different concentrations of *R. tomentosa* extract (0.2, 0.4, and 0.8 g/kg) + CCl_4_ (Black column).

In the present investigation, the administration of CCl_4_ to the animals resulted in elevation of TBARS by about 4.11 folds (blood), 1.62 folds (liver), and 2.58 folds (kidney) when compared to 1% DMSO treated animals (Figure 4). In contrast to the elevation of TBARS, a significant depletion of total GSH levels by 3.05 folds (blood, *P* < 0.001), and 4.13 folds (liver, *P* < 0.05) was observed (Tables 1, and 2). However, no significant decrease in GSH levels was observed in the kidney (Table 3). Treatment of mice with *R. tomentosa* leaves extract for 14 days, dose dependently restored the TBARS levels and GSH contents in blood, liver, and kidney samples, comparable to those in 1% DMSO treated animals. Similarly, *α*-tocopherol (100 mg/kg body weight) administration also prevented the formation of TBARS and maintained the GSH levels.

Free radical scavenging enzymes such as SOD, CAT, and GPx are the cellular defense enzymes against oxidative injury, decomposing superoxide, and peroxide before their interaction to form the more reactive hydroxyl radicals [34]. The activities of SOD, CAT, and GPx in blood, liver, and kidneys of all experimental mice were represented in Tables 1, 2, and 3, respectively. Several *in vivo* studies have demonstrated that exposure to CCl_4_ results in greater production of reactive oxygen species leading to imbalance in the oxidant/antioxidant status; have been manifested as lipid peroxidation and protein oxidation, and also reported that medicinal plant extracts are able to provide protection against the deleterious effects caused by free radicals [35, 36]. In all organs of CCl_4_ administrated mice, a significant depletion in CAT and GPx enzyme activities was observed, compared with 1% DMSO treated animals. A significant decrease in SOD activity was observed in the blood of CCl_4_-treated animals, but the decrease was not significant in liver and kidney tissues. *R. tomentosa* extract exhibited protective effects against CCl_4_-induced decrease in SOD, CAT and GPx enzyme activities in blood, liver, and kidneys (Tables 1, 2, and 3). At the lower doses (0.2, and 0.4 g/kg body weight), the recovery in enzyme activities was not significant, while with higher dose of extract (0.8 g/kg body weight), the recovery of enzyme activities were significant (*P* < 0.05) when compared to the values obtained from 1% DMSO treated mice. Alpha-tocopherol, treatment to the CCl_4_-injected mice, prevented the change in SOD, CAT, and GPx activities of all samples (Tables 1, 2, and 3). The protective effect of *R. tomentosa* at the dose of 0.8 g/kg body weight, against CCl_4_ induced oxidative damage was similar to the effect of *α*-tocopherol (0.1 g/kg body weight). From the results of the present study, it was observed that the order of the tissues that are affected by the oxidative stress of CCl_4_ on its biomolecules was, blood > liver > kidney.

The elevation of lipid peroxidation and depletion of endogenous antioxidants in CCl_4_ induced mice may be due to the free radicals generated during the biotransformation of CCl_4_. Other workers have reported that *R. tomentosa* at 0.4 g/kg body weight can effectively prevent the acetic acid-induced gastric ulcers *via* its antioxidant activity [37]. It is suggested that, *R. tomentosa* extract has the capacity to inhibit the effect of prooxidants/oxidants by reducing them and preventing the initiation of lipid peroxidation chain reaction. Considerable amount of research reports highlights the role of the polyphenolic constituents in the higher plants as free radical scavenging agents [38–40]. We have recently reported a new flavellagic acid and phloroglucinol (rhodomyrtosone I) from an acetone extract of *R. tomentosa* leaves [41], in addition to previous reports on acylphloroglucinols such as rhodomyrtosones A-D, rhodomyrtone, and some other known polyphenolic constituents including combretol, 3,3′,4-tri-*O*-methylellagic acid, (6R,7E,9R)-9-hydroxy-4,7-megastigmadien-3-one and *α*-tocopherol [42]. The presence of the identified phytochemicals may be responsible for the potent antioxidant activity of *R. tomentosa* leaf extract.

## 4. Conclusion

Both the *in vitro* and the *in vivo* results of the study indicated that *R. tomentosa* extract can act as a potent antioxidant. Some of the ethnomedical and reported biological activities of this plant are due its antioxidant property. Hence, it suggests that *R. tomentosa* extract can be employed as an antioxidant supplement in food products to prevent oxidation of food and also as alternative antioxidant therapeutics in the pharmacological industry to prevent the free radical damage of biomolecules that occurs in numerous human diseases. However, further investigations are needed to evaluate the toxicity and antioxidant activity of each individual phytochemical of the extract to develop it into full-fledged antioxidant supplement/therapeutics.

## Figures and Tables

**Table 1:** Antioxidants status in the blood of mice in each group.

Treatment groups	Level of nonenzymic antioxidant (Total glutathione, *μ*M/mL)	Level of antioxidant enzymes (U/mL)		
		Superoxide dismutase	Catalase	Glutathione peroxidase
1% DMSO	4.12 ± 0.11^b^	5.57 ± 0.21^c^	10.94 ± 0.87^c^	12.45 ± 0.47^c^
30% CCl_4_	1.35 ± 0.17^a^	3.02 ± 0.22^a^	7.45 ± 0.48^a^	6.57 ± 1.20^a^
*α*-tocopherol + CCl_4_	3.69 ± 0.82^c^	4.44 ± 0.41^c^	10.14 ± 0.78^c^	11.16 ± 0.99
Extract (0.2 g/kg) + CCl_4_	1.70 ± 0.82	4.30 ± 0.15	9.24 ± 0.46^ac^	7.64 ± 1.71^a^
Extract (0.4 g/kg) + CCl_4_	3.06 ± 0.80	4.33 ± 0.65	10.02 ± 0.41^c^	9.46 ± 0.24
Extract (0.8 g/kg) + CCl_4_	3.94 ± 0.10^b^	4.94 ± 0.36^c^	10.49 ± 0.48^c^	10.64 ± 1.13^c^

Values are mean ± SEM; ^a^ *P* < 0.01 versus 1% DMSO-treated group (*n* = 6); ^b^ *P* < 0.01 versus CCl_4_-treated group (*n* = 6); ^c^ *P* < 0.05 versus CCl_4_-treated group (*n* = 6).

**Table 2:** Antioxidant status in the liver of mice in each group.

Treatment groups	Level of nonenzymic antioxidant (Total glutathione, *μ*M/mg tissue)	Level of antioxidant enzymes (U/mg tissue)		
		Superoxide dismutase	Catalase	Glutathione peroxidase
1% DMSO	25.29 ± 9.01^b^	2.17 ± 0.42	14.68 ± 0.31^c^	2.65 ± 0.41
30% CCl_4_	6.10 ± 2.96^a^	0.68 ± 0.24	9.85 ± 0.20^a^	0.38 ± 0.06
*α*-tocopherol + CCl_4_	22.40 ± 1.03^b^	1.54 ± 0.22	14.07 ± 0.49^ac^	2.51 ± 0.30
Extract (0.2 g/kg) + CCl_4_	10.84 ± 0.66	1.02 ± 0.17	10.92 ± 0.42^a^	1.17 ± 0.18
Extract (0.4 g/kg) + CCl_4_	15.97 ± 0.80	1.05 ± 0.29	11.99 ± 0.26^c^	1.27 ± 0.23
Extract (0.8 g/kg) + CCl_4_	22.85 ± 0.65^b^	1.33 ± 0.48	13.99 ± 0.18^c^	2.22 ± 0.63

Values are mean ± SEM; ^a^ *P* < 0.01 versus 1% DMSO-treated group (*n* = 6); ^b^ *P* < 0.01 versus CCl_4_-treated group (*n* = 6); ^c^ *P* < 0.05 versus CCl_4_-treated group (*n* = 6).

**Table 3:** Antioxidant status in the kidney of mice in each group.

Treatment groups	Level of nonenzymic antioxidant (Total glutathione; *μ*M/mg tissue)	Level of antioxidant enzymes (U/mg tissue)		
		Superoxide dismutase	Catalase	Glutathione peroxidase
1% DMSO	4.89 ± 0.19	2.49 ± 0.74	11.25 ± 0.75^b^	2.35 ± 1.19^b^
30% CCl_4_	4.27 ± 0.02	1.74 ± 0.62	7.52 ± 0.10^a^	0.31 ± 0.02
*α*-tocopherol + CCl_4_	4.60 ± 0.19	2.30 ± 0.20	11.22 ± 0.30^b^	2.00 ± 0.26^b^
Extract (0.2 g/kg) + CCl_4_	4.43 ± 0.02	2.16 ± 0.61	9.01 ± 0.49	1.02 ± 0.16
Extract (0.4 g/kg) + CCl_4_	4.57 ± 0.03	2.34 ± 0.56	9.65 ± 0.30^b^	1.16 ± 0.19
Extract (0.8 g/kg) + CCl_4_	4.63 ± 0.08	2.37 ± 0.77	10.96 ± 0.21^b^	2.02 ± 0.22^b^

Values are mean ± SEM;^ a^ *P* < 0.01 versus 1% DMSO-treated group (*n* = 6); ^b^ *P* < 0.01 versus CCl_4_-treated group (*n* = 6).
